# The Effect of Glycemic Control on Endothelial and Cardiac Dysfunction Induced by Red Blood Cells in Type 2 Diabetes

**DOI:** 10.3389/fphar.2019.00861

**Published:** 2019-08-02

**Authors:** Ali Mahdi, Tong Jiao, Jiangning Yang, Oskar Kövamees, Michael Alvarsson, Maaria von Heijne, Zhichao Zhou, John Pernow

**Affiliations:** ^1^Division of Cardiology, Department of Medicine, Karolinska Institutet, Karolinska University Hospital, Stockholm, Sweden; ^2^Division of Endocrinology and Diabetology, Department of Molecular Medicine and Surgery, Karolinska Institutet, Karolinska University Hospital, Stockholm, Sweden; ^3^Department of Clinical Sciences, Karolinska Institutet, Danderyd Hospital, Danderyd, Sweden

**Keywords:** red blood cells, endothelial dysfunction, ischemia, reperfusion, diabetes, glycemic control

## Abstract

Red blood cells (RBCs) from patients with type 2 diabetes mellitus (T2DM) induce endothelial dysfunction and impair cardiac function following ischemia *via* increase in RBC arginase and oxidative stress. Here, we aimed to elucidate whether the effect of RBC-mediated cardiac impairment following ischemia and endothelial dysfunction in T2DM is dependent on glycemic control. Patients with T2DM at poor glycemic control (T2DM PGC) and at improvement in glycemic control (T2DM IGC) and healthy subjects were recruited. Isolated RBCs from subjects were incubated with aortic rings from healthy wild-type rats with subsequent evaluation of endothelium-dependent relaxation (EDR) using wire myograph. Moreover, RBCs were administered to isolated wild-type rat hearts with subsequent evaluation of left ventricular developed pressure (LVDP) during reperfusion using Langendorff setup. In separate experiments, RBCs were preincubated with an arginase inhibitor before perfusion. Blood glucose and glycated hemoglobin were 33 and 26%, respectively, lower in T2DM IGC compared with those in T2DM PGC. RBCs from T2DM PGC and T2DM IGC impaired EDR to a similar magnitude compared with RBCs from healthy subjects. LVDP was significantly impaired in hearts given RBCs from T2DM PGC as compared with those from healthy subjects. The impairment of LVDP induced by T2DM PGC was attenuated by RBCs from T2DM IGC. Arginase inhibition improved LVDP to a similar extent between T2DM PGC and IGC groups. These observations indicate that glycemic control abrogate the impairment in postischemic recovery but not endothelial dysfunction induced by RBCs from T2DM. Moreover, inhibition of RBC arginase improves cardiac function irrespective of glycemic control.

## Introduction

Type 2 diabetes mellitus (T2DM) represents a major risk factor for the development of cardiovascular disease including coronary artery disease (CAD). Despite several efforts in reducing cardiovascular morbidity and mortality with conventional glucose-lowering treatment, convincing improvements in outcomes have not been achieved (Palmer et al., 2016). More recent studies have shown that glucagon-like peptide 1 receptor agonists and sodium glucose co-transporter 2 inhibitors have a secondary preventive effect among T2DM patients at high risk for cardiovascular complications (Zinman et al., 2015; Marso et al., 2016). However, the mechanism behind these protective properties is not completely understood and seems to be related to effects beyond glucose lowering (von Lewinski et al., 2017).

It is generally considered that endothelial dysfunction, characterized by reduced bioavailability of nitric oxide (NO), is a key component behind vascular complications in T2DM. NO is formed utilizing L-arginine as substrate by the enzyme endothelial nitric oxide synthase (eNOS) (Lundberg et al., 2015). We have recently shown that arginase, an enzyme that reciprocally regulates NO formation, is central for the development of endothelial dysfunction in T2DM (Mahdi et al., 2018). Previous studies have concentrated on pathological alterations occurring in the vascular wall, but recent studies have indicated an intriguing interaction between red blood cells (RBCs) and the vascular wall in T2DM. It is well described that RBCs undergo changes in T2DM including impairment in deformability with changes in the cytoskeletal arrangement, changes in morphology, and increased adhesiveness to endothelial cells (Wautier et al., 1994; Gyawali et al., 2012; Buys et al., 2013). Of further importance, a detrimental effect of RBCs on cardiovascular function in T2DM using *ex vivo* models of cardiac and endothelial function was recently shown (Yang et al., 2018; Zhou et al., 2018). These studies identified the RBC as a key mediator in the development of endothelial dysfunction and postischemic cardiac recovery (Yang et al., 2018; Zhou et al., 2018; Pernow et al., 2019). These effects are mediated through upregulation of arginase and formation of reactive oxygen species in the RBC.

We and others have recently demonstrated that the impairment in *in vivo* endothelial function in patients with T2DM at poor glycemic control is maintained also following improvement in glycemic control (Costantino et al., 2017; Mahdi et al., 2018). In addition, the magnitude of the beneficial effect of arginase inhibition on endothelial function was unchanged after improvement in glycemic control (Mahdi et al., 2018). However, the effect of glycemic control on cardiovascular dysfunction induced by RBCs in T2DM is unclear (Pernow et al., 2019). The aim of this sub-study was, therefore, to evaluate the effect of glycemic control on endothelial dysfunction and postischemic injury induced by RBCs from T2DM patients.

## Material and Methods

### Patients and Animals

In this prespecified sub-study of a main study investigating the effect of glycemic control on endothelial function and the effect of arginase inhibition in patients with T2DM (Mahdi et al., 2018), 16 T2DM patients at poor glycemic control (T2DM PGC) and 16 T2DM patients following improvement in glycemic control (T2DM IGC) were included. Eleven healthy subjects were also included as controls. Inclusion and exclusion criteria were as previously described (Mahdi et al., 2018). Improvement in glycemic control among T2DM patients was achieved by an intensive educational program including lifestyle changes, dietary advices and equipment with glucose monitoring devices as well as optimization of medications by a specialist in diabetology.

Following an overnight fasting period, whole blood was collected in heparinized tubes with subsequent isolation of RBCs by immediate centrifugation at +4°C and 1,000g for 10 min followed by three washing cycles with Krebs–Henseleit (KH) buffer (Zhou et al., 2018).

Rats from wild-type Sprague–Dawley or Wistar rats at age 9–16 weeks (Charles River Sulzfeld, Germany) were anesthetized with pentobarbital (50 mg/kg i.p.) followed by thoracotomy and isolation of aortas or hearts.

The inclusion of patients with T2DM was performed according to the Declaration of Helsinki. All subjects were informed of the purpose and gave their oral and written informed consent. The study was approved by the regional ethical review board for studies on human subjects. Animal care and all protocols were approved by the regional ethical committee and conformed to the Guide for Care and Use of Laboratory Animals.

### RBC-Tissue Co-Incubation and Determination of Vascular Reactivity

Washed RBCs from patients with T2DM and healthy subjects were diluted to a hematocrit of ∼45% with KH buffer and incubated with aortic rings in cell culture incubator at 37°C with 5% CO_2_ for 18 h (Zhou et al., 2018). After the incubation, aortic rings were thoroughly washed and mounted in wire myographs for measurement of isometric tension as described in detail elsewhere (Zhou et al., 2018). Endothelium-dependent relaxation (EDR) was determined by application of cumulatively increasing concentrations (10^−9^–10^−5^ M) of acetylcholine (ACh) to pre-constricted vascular segments with 9,11-dideoxy-9α,11α-methanoepoxy PGF_2α_ (U46619).

### RBC Administration to Isolated Langendorff Hearts

**Figure 1:**
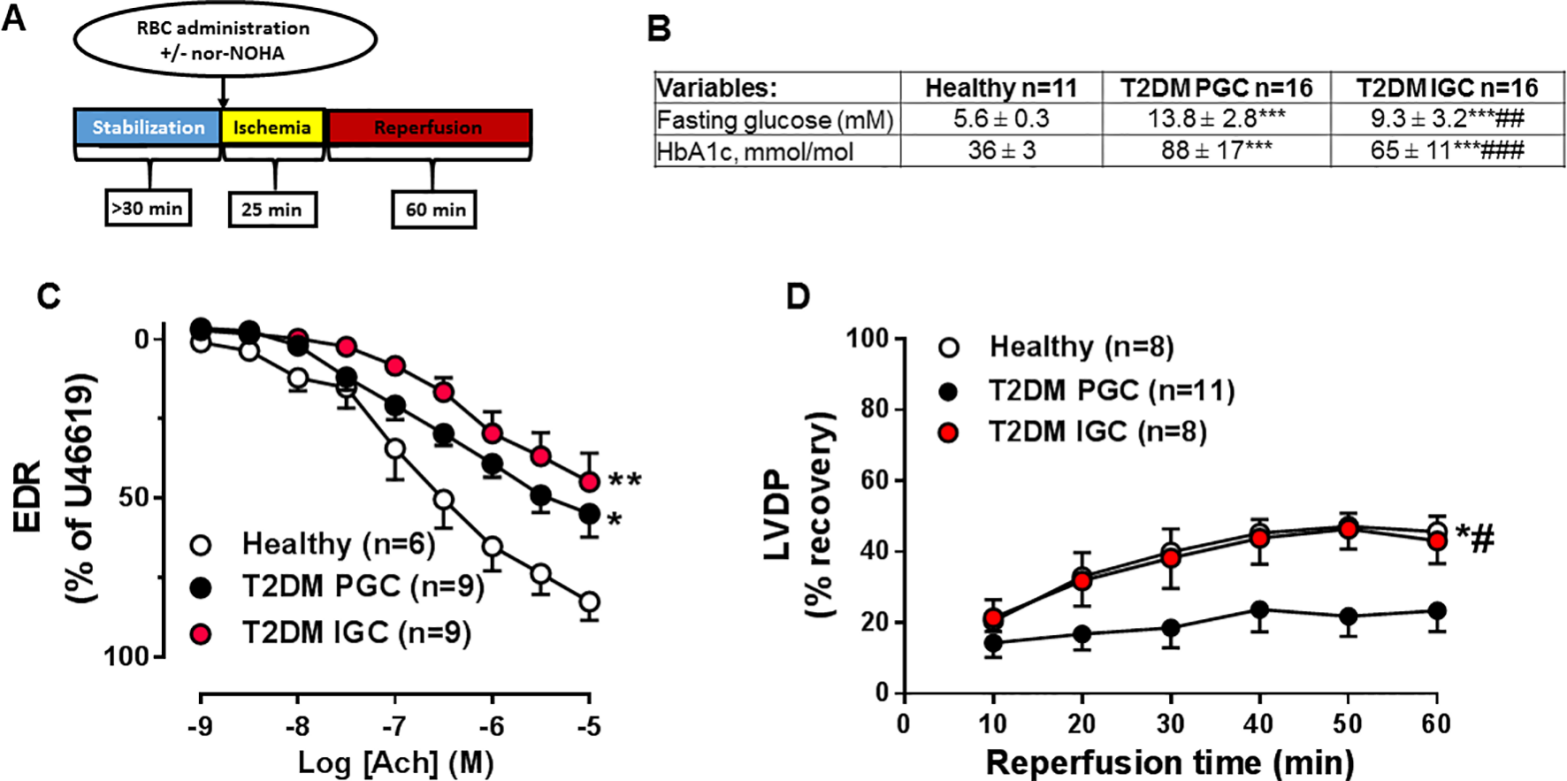
Protocol for the ischemia/reperfusion experiments in isolated hearts **(A)**. Indices of glucose **(B)** in healthy subjects and patients with type 2 diabetes mellitus (T2DM) at poor glycemic control (T2DM PGC) and improvement in glycemic control (T2DM IGC). Effect of isolated red blood cells (RBCs) from healthy subjects or patients with T2DM at PGC and IGC on endothelium-dependent relaxation (EDR, **C**) induced by acetylcholine or left ventricular developed pressure (LVDP, **D**) following ischemia. Number of observations are indicated. Values are mean ± SEM. *P < 0.05, **P < 0.01, ***p < 0.001 vs. Healthy, #*p* < 0.05, ##p<0.01, ###*p* < 0.001 vs T2DM PGC.

Isolated hearts were mounted in a Langendorff setup and perfused with KH buffer as previously described (Yang et al., 2018). A balloon-tipped catheter in the left ventricle continuously recorded left ventricular developed pressure (LVDP) (Yang et al., 2018). Following a stabilization period, hearts were subjected to 25 min of global ischemia by stopping the perfusion followed by 60-min reperfusion (**Figure 1A**). The perfusion pressure was constant throughout the experiment and set at 75 mmHg.

Immediately after the onset of ischemia, RBCs diluted to ∼30% hematocrit with KH buffer of a total volume of 3 ml were injected through a sidearm in the perfusion system, allowing the suspension to be present in the coronary circulation during the whole ischemic period (**Figure 1A**). To determine the involvement of arginase, RBCs were preincubated with the arginase inhibitor N^ω^-hydroxy-nor-L-arginine (nor-NOHA 1 mM). The experimental protocol is schematically illustrated in **Figure 1A**.

### Arginase Activity Measurements in RBCs

Arginase activity was determined as previously described by a colometric assay (Yang et al., 2018; Zhou et al., 2018). MnCl_2_ (75 μl of 10 mM) was added to lysed RBCs. Following 10 min incubation at 56°C, L-arginine (0.5 M of 50 μl) was added with subsequent incubation at 37°C for 60 min. The reaction was stopped by the addition of 400-μl stop solution (H_2_SO_4_:H_3_PO_4_:H_2_O 1:3:7). After adding 25 μl of α-isonitrosopropiophenone (9% in ethanol), the mixture was incubated at 100°C for 60 min. After centrifugation for 5 min at 5,000g, the concentration of urea was determined in a spectrophotometer (Wallac 1420 VICTOR2™) at 540 nm.

### Statistical Analyses

Baseline characteristics are presented as means ± standard deviation unless otherwise stated. Differences in variables between the groups were calculated with one-way analysis of variance with Tukey’s *post hoc* test for multiple comparisons. EDR is presented as percent relaxation from preconstriction induced by U46619 during application of cumulative concentrations of ACh. LVDP is presented as percentage recovery during reperfusion from baseline. Statistical analyses for EDR and LVDP were performed with two-way analysis of variance and expressed as mean ± standard error of mean. Arginase activity was calculated by paired *t*-test. *n* refers to the number of animal and number of subjects included in each group. One animal was used for each subject recruited. All statistical analyses were performed using Prism 7.0, GraphPad, San Diego, CA, USA. *P* < 0.05 was considered as statistically significant.

## Results

### Subjects

Baseline clinical characteristics and medication of the study subjects are presented in **Figure 1B** and **Table 1**. Indices of glucose control were markedly better in patients with T2DM IGC compared with those with T2DM PGC, with a reduction in fasting blood glucose and glycated hemoglobin (HbA1c) by 33 and 26%, respectively (**Figure 1B**). T2DM IGC had been on glucose-lowering intervention for 17 ± 5 weeks (mean ± SD). We previously showed that *in vivo* endothelium-dependent vasodilatation in the forearm circulation of this patient cohort is unaltered following such improvement in glycemic control (Mahdi et al., 2018).

**Table 1:** Characteristics of healthy subjects and type 2 diabetes mellitus (T2DM) at poor glycemic control (T2DM PGC) and improvement in glycemic control (T2DM IGC).

Variables:	Healthy *n* = 11	T2DM PGC *n* = 16	T2DM IGC *n* = 16
Age	60 ± 8	64 ± 10	64 ± 10
No. of males	9	12	12
Years since diagnosis	−	13 ± 9	13 ± 9
BMI, kg/m^2^	25.6 ± 1.7	31.3 ± 4.3***	31.3 ± 4.4**
BP (mmHg):			
Systolic	135 ± 13	144 ± 19	147 ± 21
Diastolic	82 ± 8	84 ± 14	82 ± 10
No. of smokers	0	5	5
Hemoglobin, g/L	148 ± 10	142 ± 15	137 ± 16
Creatinine, mmol/L	84 ± 13	81 ± 19	84 ± 21
Triglycerides, mmol/L	1.2 ± 0.4	2.1 ± 0.9*	1.6 ± 0.8
Total cholesterol, mmol/L	5.2 ± 1.0	4.3 ± 1.2	3.5 ± 0.7***
HDL, mmol/L	1.5 ± 0.4	1.1 ± 0.3*	1.1 ± 0.3*
LDL, mmol/L	3.2 ± 0.9	2.2 ± 1.1*	1.6 ± 0.6***
General medication, *n* (%)			
ACEi/ARB	−	9	12
Aspirin	−	6	6
Lipid lowering	−	12	13
b-blockers	−	7	7
Calcium channel i	−	5	6
Glucose lowering medication, *n*			
Insulin	−	8	10
Metformin	−	14	14
GLP1	−	4	9
DPP-4i	−	4	4
SU	−	4	5
SGLT2i	−	1	2

ACEi, angiotensin-converting enzyme; ARB, angiotensin II receptor blocker; BMI, body mass index; DPP-4i, dipeptidyl peptidase-4 inhibitor; GLP1, glucagon like peptide 1 analogue; HbA1c, glycated hemoglobin; HDL; high-density lipoprotein; LDL, low-density lipoprotein; SGLT2i, sodium-glucose cotransporter 2 inhibitor. *p<0.05,**p < 0.01, ***p < 0.001 vs healthy. Data are presented as means ± standard deviation.

### Effects of Glycemic Control on Endothelial Dysfunction and Postischemic Injury Induced by RBCs From T2DM Patients

EDR was significantly impaired in aortas incubated with RBCs from T2DM in comparison with RBCs from healthy control subjects (**Figure 1C**). There was no difference in the degree of impairment of EDR induced by RBCs from patients with T2DM IGC and T2DM PGC (**Figure 1C**). A separate analysis of a subgroup of paired observations using RBCs from the same subjects before and after glucose-lowering intervention showed a similar trend (**S. Fig 1A**). This indicates that glycemic control does not affect the negative effect of RBCs from patients with T2DM on endothelial function.

Recovery of LVDP following global myocardial ischemia was impaired in hearts given RBCs from T2DM PGC in comparison with hearts given RBCs from healthy controls. By contrast, the recovery of LVDP was significantly improved when RBCs from T2DM IGC was given to the isolated heart and not different from that observed when giving RBCs from healthy subjects (**Figure 1D**). A separate analysis of a subgroup of paired observations using RBCs from the same individuals before and after glucose-lowering intervention was performed and showed a similar improvement in LVPD after improvement on glycemic control (**S. Fig 1B**). These data indicate that the effect of RBCs from patients with T2DM on postischemic recovery is dependent on glycemic control.

### Role of Arginase in Postischemic Recovery Mediated by RBCs From Patients With T2DM Following Glycemic Control

**Figure 2:**
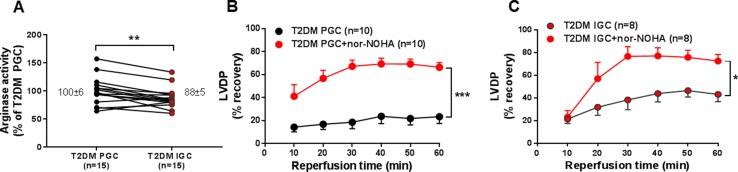
Arginase activity in RBCs from T2DM patients at PGC and IGC **(A)**. Effect of RBCs from patients with T2DM with or without preincubation with nor-NOHA on LVDP at PGC **(B)** and IGC **(C)**. Numbers of observations are indicated. Values are mean ± SEM. *p < 0.05, **p < 0.01, ***p < 0.001.

Others and we previously reported that arginase activity is increased in RBCs from patients with T2DM (Ramirez-Zamora et al., 2013; Zhou et al., 2018). Here, we further evaluated whether arginase levels are dependent on glycemic control by measuring arginase activity in RBCs. A significant reduction in arginase activity following improvement of glucose indices was observed (**Figure 2A**), indicating that arginase activity is dependent on the degree of glycemic control.

Based on the observation that LVDP, but not EDR, was improved by RBCs from patients with T2DM IGC, we investigated the role of RBC arginase. A previous study has demonstrated that RBC arginase is a critical regulator of RBC NO bioactivity and tolerance to myocardial ischemia–reperfusion (Yang et al., 2013). Inhibition of RBC arginase from T2DM patients with nor-NOHA significantly improved the recovery of LVDP to similar levels at both PGC (**Figure 2B**) and IGC (**Figure 2C**). This suggests that improvement in postischemic recovery by arginase inhibition is effective also following glycemic control.

## Discussion

The present study reports three major findings in light of the recently described interaction between RBCs from patients with T2DM and the cardiovascular system (Pernow et al., 2019). Firstly, the detrimental effects of RBCs from patients with T2DM on cardiac ischemia–reperfusion injury were markedly attenuated following improvement in glycemic control. Arginase inhibition induced protection against cardiac ischemia–reperfusion injury *via* a mechanism that is unrelated to glycemic control. Secondly, in contrast to the effect on cardiac function, our results indicate that endothelial dysfunction induced by RBCs from patients with T2DM was maintained even following glucose optimization. Thirdly, arginase activity is dependent on the degree of glycemic control. These observations suggest that there is a disparity on the effects of dysfunctional RBCs in T2DM following glycemic control across the cardiovascular system.

Glucose-lowering therapy in clinical studies assessing major cardiovascular events and cardiovascular death has not been successful (Palmer et al., 2016). Previous clinical trials that have focused on the effect of glucose lowering on the risk for future cardiovascular events and endothelial dysfunction represent a risk factor for such development (Xu et al., 2014). It is, therefore, expected that endothelial dysfunction induced by RBCs from patients with T2DM was not attenuated by glucose-lowering interventions. This may support the hypothesis that RBCs play an important role in the regulation of vascular function in T2DM.

Of interest is the observation that the postischemic recovery of isolated hearts given RBCs from patients with T2DM following glucose-lowering interventions is markedly improved. Accordingly, RBC arginase activity following glycemic control was reduced, which may suggest that the improvement in postischemic recovery could partially be explained by this reduction. Contrary to these observations, the reduction in arginase following glycemic control did not improve endothelial dysfunction induced by RBCs from T2DM. RBCs are more susceptible to oxidative insult induced by hypoxia during ischemia (Huertas et al., 2013). In fact, it has been suggested that formation and export of ROS from RBCs are increased during hypoxic conditions (Rifkind et al., 2018). There are also indications that glycemic control among patients with T2DM influences oxygen uptake, which may further affect the oxidative balance (Verges et al., 2015). As arginase regulates reactive oxygen species (ROS) formation the oxidative insult may be attenuated following glycemic control as a consequence of reduction in arginase activity, which is of importance in the ischemia-reperfusion setting.

One of the mechanisms by which RBCs exerts its vasoactive effects is through hypoxic vasodilatation mediated through export of NO bioactivity (McMahon et al., 2002). Since the global ischemic heart model is a severe model of hypoxia, it may be speculated that this mechanism could be dependent on glycemic control. This is supported by the fact that arginase is reduced following glycemic control. Consequently, this may lead to increase in export of NO bioactivity in situations of improved glycemic control resulting in better postischemic recovery. Collectively, the results from the current study point to that export of NO bioactivity from RBCs in the setting of myocardial ischemia may be of greater importance than regulation of endothelial dysfunction by RBCs under normoxic conditions in T2DM.

Interestingly, arginase inhibition markedly improved postischemic recovery in the presence of RBCs from T2DM patients both before and after glycemic control. We previously demonstrated that glucose induce a modest but significant increase in RBC arginase activity (Zhou et al., 2018). In the current study, we observe that this upregulation is partly reversible by long-term glycemic control. Ischemia could also possibly modulate arginase and ROS in RBCs (Rifkind et al., 2018), which may be related to glycemic control. Future studies are warranted to elucidate the exact mechanisms underlying the involvement of glycemic control in the effect of RBCs from T2DM on postischemic recovery. Nevertheless, the present data suggest that arginase inhibition provides protection against the detrimental effects of RBCs from patients with T2DM beyond that obtained with glucose-lowering therapy.

Improved glycemic control reduced arginase activity but failed to affect endothelial dysfunction induced by RBCs from patients with T2DM. Since we previously observed an attenuation in T2DM-RBC-induced endothelial dysfunction by arginase inhibition (Zhou et al., 2018), this may indicate that pharmacological inhibition may reduce arginase activity even more than that obtained by glycemic control. The magnitude of inhibition achieved by pharmacological agents may be required to suppress ROS and to rescue endothelial function, as we previously observed an attenuation in ROS production by arginase inhibition in RBCs from patients with T2DM (Zhou et al., 2018). Other mechanisms, not strictly limited to arginase and NO, may also be involved in the sustained endothelial dysfunction induced by RBCs from T2DM following glycemic control. These mechanisms may involve changes similar to those observed in hyperglycemic memory such as sustained formation of advanced glycation products (AGEs) and their receptors (RAGEs). Indeed, RBCs from T2DM are known to induce changes in endothelial AGE-RAGE (Wautier et al., 1981). Whether these changes are of functional importance in glycemic control remains to be elucidated.

As mentioned, glycemic control has not convincingly shown a benefit in the development of cardiovascular complications and mortality. The role of glycemic control during acute myocardial infarction and the subsequent complications in the clinical setting is different, however, and poorly characterized (Sasso et al., 2018). Increased levels of glucose at admission in patients with T2DM are associated with greater mortality risk (Kosiborod et al., 2005). In line with this, among patients referred to a cardiac intensive care unit, mortality was increased in those with high glucose levels at admission and during hospitalization, irrespective of if the patients had T2DM or not (Lipton et al., 2013). Based on the results of the current study, it is tempting to speculate that one of the underlying mechanisms behind these observations may be related to improvement in RBC function following glycemic control during myocardial ischemia.

A limitation in the current study is that recovery of cardiac function following ischemia rather than infarct size was determined. Our previous studies indicate that impairment in cardiac recovery induced by RBCs in T2DM reflects increased infarct size (Yang et al., 2018). These data support the notion that the differences we observe in functional parameters (LVDP) reflect differences in infarct size using the present experimental approach.

In conclusion, the present study demonstrates that the detrimental effects of RBCs from patients with T2DM on postischemic recovery but not endothelial function is markedly attenuated following improved glycemic control. Furthermore, inhibition of RBC arginase improves postischemic recovery beyond that achieved with glycemic control. These findings advance our understanding regarding the important regulation of cardiovascular function by RBCs in T2DM.

## Data Availability

The raw data supporting the conclusions of this manuscript will be made available by the authors, without undue reservation, to any qualified researcher

## Ethics Statement

This study was carried out in accordance with the recommendations of Declaration of Helsinki with written informed consent from all subjects. All subjects gave written informed consent in accordance with the Declaration of Helsinki. The protocol was approved by the regional ethical review board in Stockholm. This study was carried out in accordance with the recommendations of Guide for Care and Use of Laboratory Animals published by the U.S. National Institutes of Health. The protocol was approved by the regional ethical committee.

## Author Contributions

AM, TJ, JY, OK, and ZZ designed, performed the experiments, or analyzed the data. AM, JY, OK, MA, MvH, ZZ, and JP conceived the study. AM wrote the manuscript. All authors participated in the interpretation of the data and provided critical review of the manuscript.

## Funding

This work was supported by the Swedish Research Council [2016–01284], the Swedish Heart and Lung Foundation [20160239], the Stockholm County Council (ALF) [20160084], Karolinska Institutet/Stockholm County Council Strategic Cardiovascular Programme, Söderberg Foundation [M60/15], the European Foundation for the Study of Diabetes, the Diabetes Research and Wellness Foundation [720-1519-16 and 363-PG], and Karolinska Institutet.

## Conflict of Interest Statement

The authors declare that the research was conducted in the absence of any commercial or financial relationships that could be construed as a potential conflict of interest.

## Supplementary Material

Figure S1 Effect of RBCs from the same patients with T2DM at poor glycemic control (T2DM PGC) and following improvement in glycemic control (T2DM IGC) on EDR **(A)** and LVDP **(B)**. Number of observations are indicated. Values are mean ± SEM. **p < 0.01 vs T2DM PGC.
