# Supplementary figures and images for: The Effect of Glycemic Control on Endothelial and Cardiac Dysfunction Induced by Red Blood Cells in Type 2 Diabetes
Source: Front Pharmacol. 2019 Aug 2;10:861. doi: 10.3389/fphar.2019.00861 (PMC6688094; doi:10.3389/fphar.2019.00861)

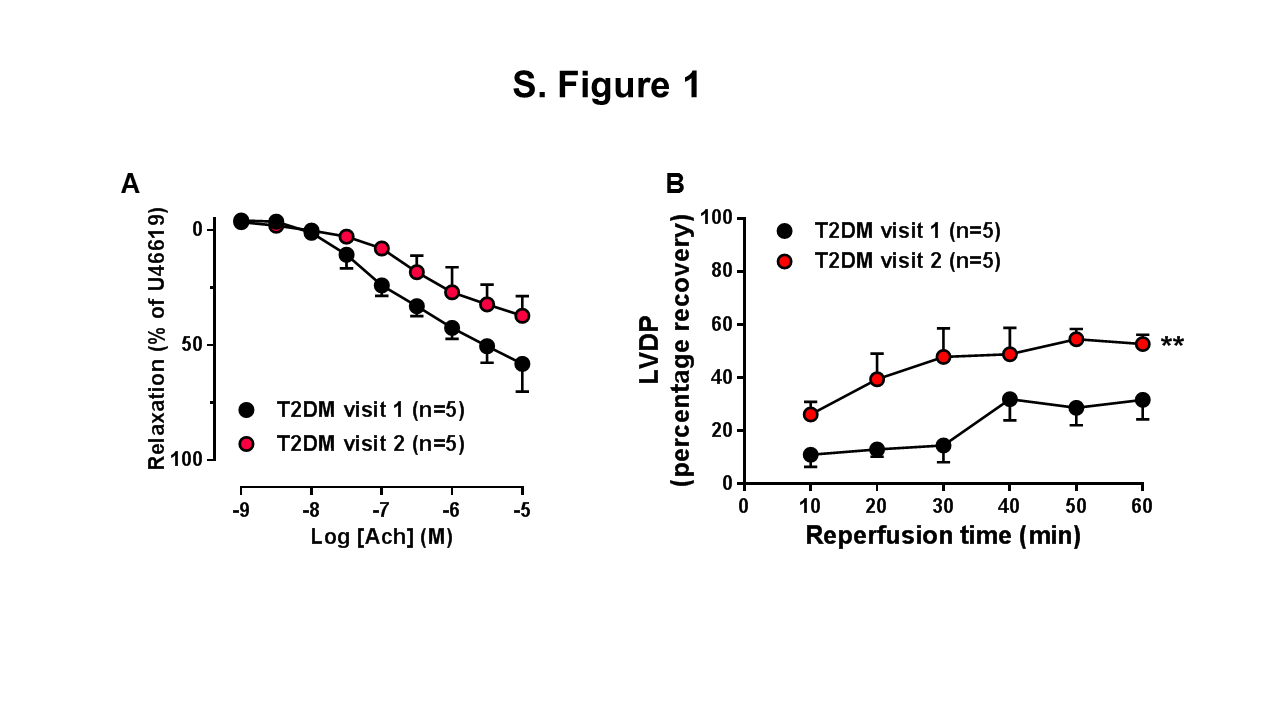

Supplement: Figure S1 — Effect of RBCs from the same patients with T2DM at poor glycemic control (T2DM PGC) and following improvement in glycemic control (T2DM IGC) on EDR (A) and LVDP (B). Number of observations are indicated. Values are mean ± SEM. **p < 0.01 vs T2DM PGC. [file Image_1.tif]
